# Anterior-to-Posterior Epidural Migration of a Lumbar Disc Herniation at L1-L2: A Case Report

**DOI:** 10.7759/cureus.27568

**Published:** 2022-08-01

**Authors:** Hamisi M Mraja, Ozcan Kaya, Tural Mammadov, Selhan Karadereler, Azmi Hamzaoglu

**Affiliations:** 1 Istanbul Spine Center, Istanbul Florence Nightingale Hospital, Istanbul, TUR; 2 Orthopaedics and Traumatology, Istanbul Kanuni Sultan Suleyman Training and Research Hospital, Istanbul, TUR; 3 Radiology, Istanbul Florence Nightingale Hospital, Istanbul, TUR

**Keywords:** spinal decompression, microsurgical, epidural ligaments, disc herniation, posterior epidural migration

## Abstract

Protruded disc fragments that penetrate the posterior longitudinal ligament (PLL) migrate rostral or caudal in the vertical plane, some laterally in the horizontal plane, or into the foramina involving the anterior aspect of the spinal canal. Often, there is migration to the ventral epidural space. However, posterior epidural migration of a lumbar disc herniation (PEMLDH) is a rare phenomenon that makes the differential diagnosis challenging. We describe a rare case of anterior-to-posterior epidural migration of a lumbar disc herniation at the L1-L2 level. It was treated microsurgically after total laminectomy, and total resection of the lesion was carried out. PEMLDH is a unique condition causing neurological deficits at different levels. Due to different localization of disc herniations, optimal diagnosis becomes critical for determining the timing and type of treatment surgically.

## Introduction

Disc herniation is the displacement of disc elements through a disruption in the annulus fibrosis past the border of the intervertebral disc space [1]. Two anatomical layers are involved in developing the intervertebral disc sequestration: the annulus fibrosus of the intervertebral disc and the posterior longitudinal ligament (PLL). Also, epidural ligaments (ELs of Hofmann) have been described as anatomical factors preventing the posterolateral migration of herniated disc fragments. Different anatomic variations have been described, including anterior, lateral, or posterior to the spinal dura [2]. Generally, this condition occurs due to age-related degeneration of the disc, failure of nutrient supply, mechanical loading of the spine, and genetic factors.

Lumbar disc herniation (LDH) usually migrates rostral or caudal in the vertical plane. Some LDHs migrate in the horizontal plane laterally or the foramina involving the anterior aspect of the spinal canal [1]. Also, 35%-72% of all LDHs are associated with fragment migration [3]. The migration of extruded lumbar disc materials exhibits specific patterns. Free fragments can migrate anywhere within the epidural space or even be located inside the dura mater. However, anatomical barriers limit their migration, especially to the posterior epidural and/or intradural spaces where isolated disc fragments are exceptionally found.

PEMLDH is an absolutely rare entity, and up to now, it has been only exceptionally reported in the literature [4]. Sengoz et al. reported a 0.27% incidence among 2880 patients [5]. Frequently PEMLDH is seen in adults averaging 53 years old, with a male predominance [6].

Treatment choice depends mainly on the radiological and clinical status of the patients [7]. Moreover, tumors located in the posterior epidural space may resemble PEMLDH radiologically and clinically. Hence, it is challenging in the differential diagnosis.

Magnetic resonance imaging (MRI) is the best modality for visualizing intraspinal canal structures, especially disc material and nerve roots [8]. Knowing the exact location of the migrated lumbar disc fragments is crucial in selecting the treatment plan.

In this report, we present a case of unusual anterior to posterior lumbar disc herniation.

## Case presentation

Medical history and physical examination

A 67-year-old male was admitted to our hospital with a history of almost one-month-old low back pain (visual analog scale 8; VAS-8). Oswestry Disability Index (ODI) was 10. Recently, he developed bilateral lower extremity paresthesia mostly on the left leg. Within one month before presentation, the patient had sudden onset of left leg numbness without any history of trauma. In his physical exam, a manual muscle test of the left and right ankle dorsiflexion strength was grade four and five, respectively. In both low extremities, provocative tests, i.e. straight leg raise (SLR; Lasegue's sign), contralateral SLR, and femoral nerve stretch test (Wasserman sign) were negative. No pathologic reflexes were identified. There was no alteration in his blood tests.

Radiological studies

**Figure 1:**
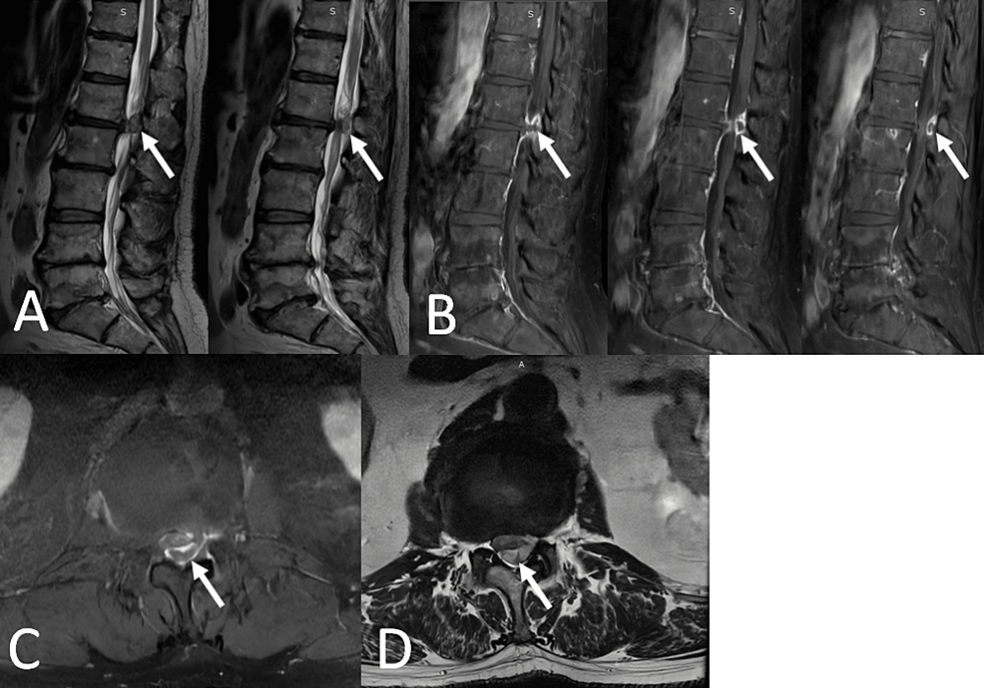
(A) Sagittal T2-weighted images demonstrate a large mass in the L1-L2 epidural space. (B) Gadolinium-enhanced sagittal T1-weighted images show a peripherally enhancing lesion in the L1-L2 epidural space that is elongating posterolaterally. (C) Gadolinium-enhanced axial T1-weighted images demonstrate a peripherally enhancing lesion (arrow) located in the anterior-to-posterior epidural space at the L1–L2 level. (D) The same lesion is seen in the T2-weighted images.

CT scan of the lumbar spine revealed a degenerative disc disease in the lumbar spine. Magnetic resonance images (MRI) of the lumbosacral showed a lesion in the posterior epidural space (16X8X12 mm), causing compression to the dural sac at the L1-L2 level (Figure 1). This posterior L1-L2 fragment was an incidental finding. Radiologically, comprehensive differential diagnoses were made, evaluating a tumor, facet cyst, epidural abscess, hematoma, and disc. Also, lumbar spinal stenosis and L2-L3, L3-L4, L4-L5, and L5-S1 disc degeneration were seen, which explained the patient’s symptoms. However, the L1-L2 disc fragment was not the main pathology explaining the patient’s symptoms. Eventually, MRI evaluation facilitated the diagnosis of this lesion as a PEMLDH. Based on the patient’s neurologic deficit and radiological evaluations, surgical intervention was decided.

Electromyography (EMG) evaluation

The left L3 and L4 roots were diagnosed with chronic period involvement and partial axonal injury.

Surgical procedure and outcome

**Figure 2:**
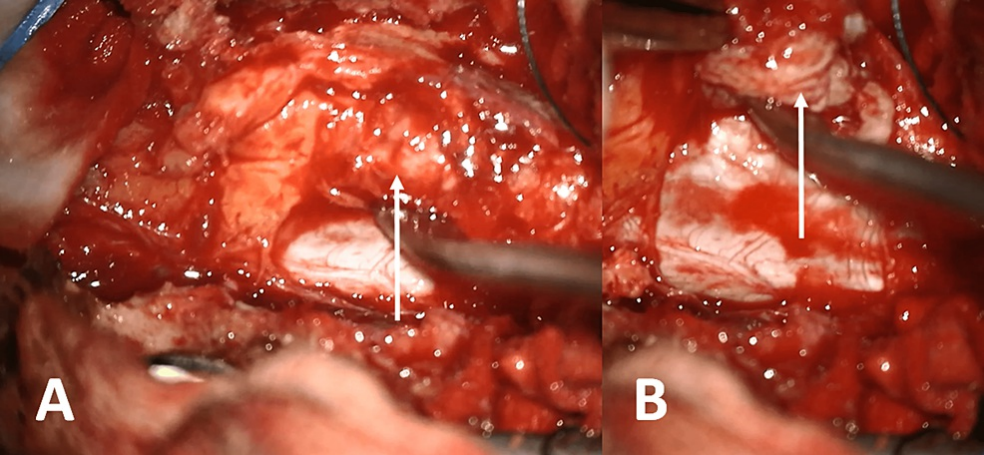
A. Intraoperative picture after total laminectomy showing a disc material located at the posterior epidural space (arrow). B. Showing the dura capsule after the removal of the lesion.

Traditionally, a posterior approach surgery was performed. T10 to pelvic posterior instrumentation was performed. Due to the lumbar spinal stenosis at theL1-L5 level and foraminal stenosis, a total laminectomy was performed at the L1-L5 level. At the L1-L2 level, after flavectomy, an atypical extruded disc was seen in the posterior space of the epidural space (Figure 2A).

**Figure 3:**
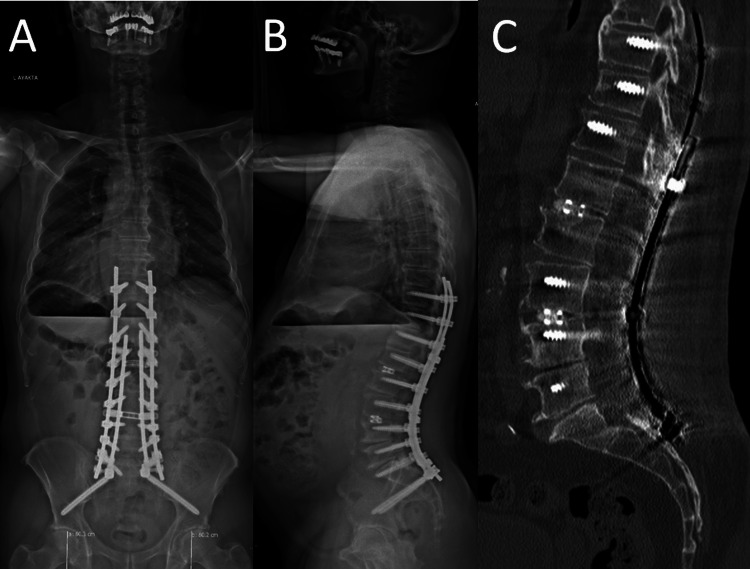
(A) Postoperative X-ray demonstrating T10-S2 posterior instrumentation. (B) Postoperative lateral X-ray demonstrating T10-S2 posterior instrumentation, preferred sagittal contoured rods, and interbody fusion of L2-L3 and L4-L5 levels. (C) Postoperative sagittal computed tomography (CT) scan demonstrating L1-L5 total laminectomy and interbody fusion of L2-L3 and L4-L5 levels.

Moreover, the lesion was also elongated to the lateral corner of the epidural space, making it surround the dura laterally. We performed a precise control under a microscope, and the lesion had no communication with the facet joint laterally. However, under the microscope, the lesion was seen to be minimally attached to the dura. Microsurgically, the lesion was totally resected (Figure 2B). Bilateral L1, L2, L3, L4, L5, and S1 foraminotomy was performed. A T10-S2 level posterior instrumentation was performed. Additionally, transforaminal lumbar interbody fusion (TLIF) was performed at the L2-L3 and L4-L5 levels (Figure 3).

**Figure 4:**
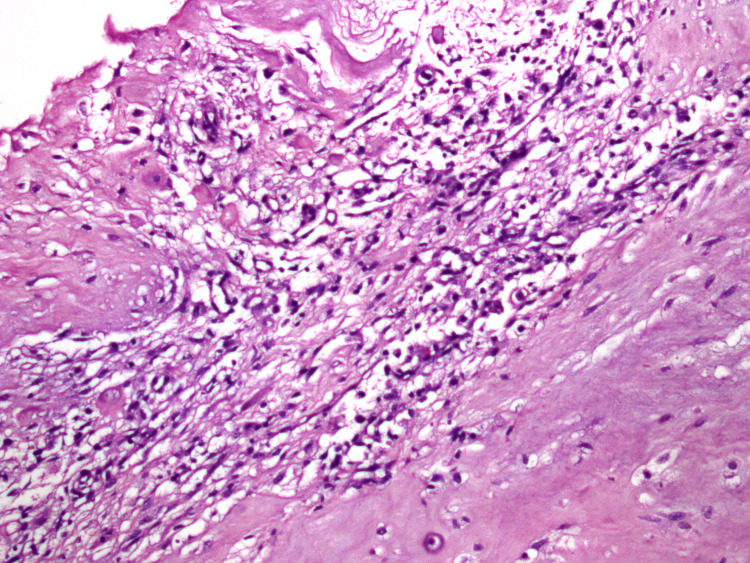
Histopathologic image findings show degenerated fibrocartilage (H&E, ×200)

Postoperatively, the neurologic deficit of the patient gradually improved. His left and right ankle dorsiflexion strength improved to grade 5 within one month. We performed a histopathologic evaluation of the lesion, which was confirmed to be a degenerative disc (Figure 4).

## Discussion

Extruded disc herniation develops after the disruption of two layers: the annulus fibrosus of the intervertebral disc and the posterior longitudinal ligament (PLL) [1]. Historically, epidural ligaments (ELs of Hofmann) have been also described as preventing the posterolateral migration of disc herniation. They were initially described as only localized ventrally to the nerve roots below the L1 spinal level [9-10]. Their variation has been further investigated demonstrating variability in most spinal levels and circumferentially interconnecting with the spinal dura [9,11]. Different anatomic variation has been described, including anterior, lateral, or posterior to the spinal dura [2]. The posterior epidural ligaments were evaluated in a cadaveric study with a 52.9% and 35.7% incidence in the lumbar and thoracic spine [9]. This minor incidence defines the rare migration of epidural herniation to the posterolateral space.

The variable clinical presentation in PEMLDH ranges from low back pain without neurologic deficits to cauda equina syndrome such as radiating pain in the legs, areflexia, sensory and motor disturbances, and bladder/bowel dysfunction [12]. Most patients present with acute cauda equina syndrome [3]. The period between symptom onset and surgery was a major factor affecting postoperative improvement [5].

Radiological findings are essential to establish the diagnosis of PEMLDH. Apart from calcified disc herniations, computed tomography (CT) is insufficient in the diagnosis of disc herniations compared to MRI [12]. MRI is considered the gold standard for diagnosis, and contrast-enhanced MRI promotes the differential diagnosis of posterior epidural lesions [12-13].

In the T1W1 MRI, herniated discs commonly exhibit an isointense signal and 80% produce a high signal on T2WI [13], and most disc fragments show peripheral rim enhancement on T1WI after gadolinium DTPA injection. Traditionally, among the radiological evaluations included in the differential diagnosis, T2WI images are more important than T1WI images. In our patient, based on the T1-weighted and gadolinium DTPA-enhanced images, our MRI findings suggested a compressed dura from a posterior epidural mass running antero-to-posterior that is surrounded by hyperintense fat tissue on T1WI [5].

PEMLDH may appear similar to the MRI findings of epidural abscess. In an abscess, there may be increased signals in the epidural fat and irregularity in the endplate margins on T2WI [4]. However, hematoma, seen after trauma, shows no rim enhancement on contrast-enhanced images [5].

The most commonly seen type in spinal cord tumors is an extradural tumor. The majority of these extradural tumors are metastases [14]. Even though just 0.15%-4% of all symptomatic disc herniation occurs in the thoracic spine [13], metastatic epidural tumors are essentially seen in this region (60%-70%), succeeded by the lumbosacral (20%-25%) and cervical spine (10%-15%) [15].

PEMLDH diagnosed radiologically and clinically in patients with neurological symptoms has to be immediately resected traditionally using a routine total laminectomy or hemilaminectomy. Recently, with minimal invasive methods, endoscopic discectomy can be performed with comprehensive decompression [16]. Early intervention usually has the best prognosis [3]. Total resection of the extruded disc is critical. In the presence of adhesions, a comprehensive evaluation should be done prior to the resection. In addition, cautious dissection of the lesion is mandatory.

## Conclusions

Posterior epidural migration of a lumbar disc herniation (PEMLDH) is a condition with low incidence. Moreover, anterior-to-posterior migration of the herniated disc material is even rarer, making it more challenging in the differential diagnosis. Comprehensive patient history and clinical, and radiological evaluation provide the most appropriate diagnosis, which is critical in choosing appropriate management. In an emergency setting, it becomes easier to define a favorable surgical decision. Up-to-date early surgical treatment is the most favorable recommendation to optimize neurological results and reduce complications in PEMLDH patients presenting with a neurological deficit.
